# How personality traits influence impulsive buying through the sequential mediation of family dynamics and self control

**DOI:** 10.1038/s41598-025-94564-3

**Published:** 2025-05-12

**Authors:** Pei Xie, ChaoZheng Huang, Tianwei Xu, Jiawei Cui

**Affiliations:** 1 School of Psychology, Sichuan Normal University, Chengdu, 610068 China; 2 School of Teacher Education, Qiongtai Normal University, Haikou, 571127 China; 3 Key Laboratory of Child Cognition and Behavior Development of Hainan Province, Haikou, 571127 China; 4 School of Judicial Police, Gansu University of Political Science and Law, Lanzhou, 730070 China; 5 School of Psychology, Northwest Normal University, Lanzhou, 730070 China

**Keywords:** Personality traits, Self-control, Family dynamics, Impulsive buying, Social psychology, Psychology, Human behaviour

## Abstract

The present investigates how personality traits influence impulsive buying (IB) among college students, emphasizing the mediating effects of family dynamics and self-control. Using a structural equation model, we analyzed responses from 578 college students in Gansu Province, China to explore the relationship between the Big Five personality traits, family dynamics, self-control, and IB tendencies. Neuroticism and extroversion are positively associated with IB, whereas conscientiousness is negatively associated with it. Family dynamics and self-control significantly mediate the relationship between personality traits and IB. This study reveals the complex interplay between individual personality traits and external factors in IB, and suggests a focus on family-specific and self-regulatory interventions. The findings provide educators and policymakers with practical strategies aimed at strengthening family relationships and self-control to curb IB behavior among youth. Furthermore, this study offers a novel perspective on IB by integrating personality traits, family dynamics, and self-control, thereby contributing valuable insights to the literature on consumer behavior as well as strategies to mitigate financial risk among young consumers.

## Introduction

With the rapid development of the economy and improvement in material living standards—especially the rise of online shopping—impulsive buying (IB) behavior has become increasingly prevalent. IB is an unplanned reaction that generates a need to make, or stimulates the potential desire to make, a purchase^1,2^. Unlike deliberate or planned purchases, IB disrupts normal consumer decision-making patterns; consumers often grapple with their desire to make a purchase and harness their willpower to stop themselves from doing so^3^. Consumers tend to overlook the utility and necessity of products in favor of the pleasure derived from buying them. While impulsive consumers may experience significant excitement and pleasure, this short-term joy leads to long-term ramifications such as debt, poor credit, increased negative emotional experiences, and gradually reduced self-esteem^4^. Notably, college students have become a major group prone to IB, leading to adverse outcomes^5^.

IB behavior is influenced by external factors such as social media and product promotions^6,7^; internal factors are generally related to individual traits and conditions. IB is also influenced by personality traits^8^. Neuroticism and extroversion are positively associated with IB, while conscientiousness is negatively correlated with it^8^. As a problematic behavior, IB is affected by internal factors such as self-control; external environmental factors—especially family factors—also influence it^9^.

Individuals with high self-control are more likely to suppress impulsive tendencies depending on the context^10^. Self-control theory posits that self-control is a key ability to suppress impulses and achieve rational decision-making^11^. Self-control directly affects IB behavior and is also influenced by personality traits and external factors, such as family dynamics^12^. As an external environmental variable, family dynamics can significantly influence psychological and behavioral development^9,13^.

Family serves as a crucial environmental factor in individual growth, substantially influencing psychological health and the cultivation of behavioral regulation^14^. Effective family dynamics provide a supportive, stable environment that fosters emotional regulation and behavioral control, which are exacerbated by insufficient family dynamics, thereby increasing the risk of IB^15^. Family dynamics lay the groundwork for the development of self-control by influencing emotional stability and behavioral habits; self-control plays a regulatory role in managing responses to consumption-related stimuli^16^. Hence, incorporating family dynamics and self-control into the relationship model between personality traits and IB behavior not only facilitates a deeper understanding of the mechanisms underlying behavioral formation but also provides a multi-dimensional, analytical framework for theoretical and empirical research. Hence, we integrated family dynamics and self-control as mediating variables to build a multiple mediation model that comprehensively analyzes the relationship between personality traits and IB behavior.

## Research hypotheses

### Hypothesis 1 (H1): personality traits significantly predict IB

Personality is a relatively stable and unique psychological and behavioral pattern formed by the interaction between innate heredity and one’s acquired environment^17^. A previous study found that in addition to genetic and environmental factors, personality (along with age and gender) explained 25% of individual differences in IB^18^. Research on the Big Five personality traits is fairly mature^19^, and previous studies have found relationships between different personality types and IB. Neuroticism and extroversion are positively correlated with IB, whereas conscientiousness is negatively correlated with it. Ratnawat and Borgave^20^ found that agreeableness and neuroticism were significantly correlated with IB behavior. Raza et al.^8^ also found that personality traits, neuroticism, and extroversion have significant, positive effects on IB. As such, we hypothesized that the Big Five personality traits would significantly predict IB behavior.

### Hypothesis 2 (H2): family dynamics play a mediating role between personality traits and IB

Family dynamics describe emotional bonds, communication patterns, and rules within a family^21^; they can influence impulsive behavior^22^. Epstein et al.^13^ suggested that family dynamics can provide certain environmental conditions for family members’ healthy growth in terms of physical, psychological, and social aspects. Individuals with good family dynamics have fewer problems such as drug abuse and risky behavior^9^. Zhang et al.^23^ found that poor family dynamics might increase gambling-related beliefs. Moreover, personality traits and family dynamics are related. A prior study revealed that neuroticism and agreeableness significantly predicted addicts’ family dynamics; greater levels of neuroticism are associated with poorer family dynamics, whereas higher levels of agreeableness are linked to better family dynamics^24^. Thus, family dynamics, as an external factor, may mediate the relationship between personality traits and impulsive behavior. Hence, we posited that family dynamics would play a mediating role in the connection between personality traits and IB.

### Hypothesis 3 (H3): Self-control plays a mediating role between personality traits and consumption

Nagar^25^ suggested that rational consumer behavior requires individuals to suppress IB through self-control, which encompasses various abilities to exert influence in the direction of one’s intentions. These abilities include the capacity to restrict as well as manage one’s own behavior, emotions, and cognitive activity; taking events and one’s physical, mental, behavioral, and external environments as the object; and aiming for the harmonious growth of society^26^. This enables individuals to make wiser, more rational choices while shopping^27^ However, when self-control is overwhelmed by desire, individuals may succumb to their emotional impulses, leading them to make a purchase. When people feel out of control, their emotions can accelerate the production of irrational and impulsive behaviors such as IB^28^. Moreover, self-control is tied to personality traits, and impulsivity is a stable personality trait that can affect IB^29^. Self-control plays a mediating role in the influence of neuroticism on IB^30^ as well as between shopping stimuli and IB behavior^10^. Thus, we hypothesized that self-control would play a mediating role between personality traits and consumption.

### Hypothesis 4 (H4): Family dynamics and self-control have a chain-mediating effect on the process of personality traits influencing IB.

**Fig. 1:**
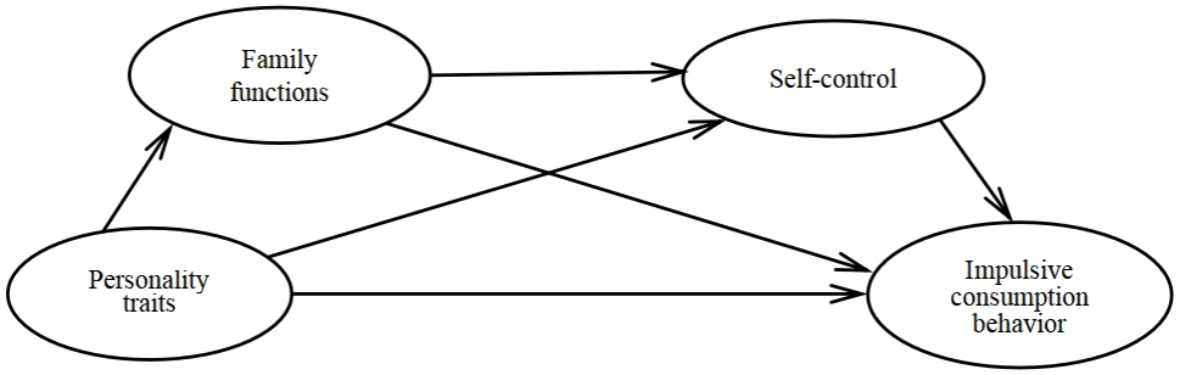
Conceptual model figure.

There are two reasons for problematic behaviors: (1) the intrinsic factors of the actor, including his/her ability, interest, hobbies, and effort; and (2) factors other than the actor, such as the growth environment and peer relationships^31^. As the main external context for individual growth, family dynamics have a substantial impact on IB behavior; self-control (as an internal factor in individual development) also has a considerable influence on IB behavior. A healthy family environment can help shape one’s personality, reduce impulsivity, and positively influence adaptation and the development of self-control^32,33^. Thus, we posited that family dynamics and self-control would have a chain-mediating effect on the process of personality traits influencing IB (see Fig. 1).

## Method

### Participants and procedure

We employed stratified random sampling to administer a questionnaire to college students in Gansu Province, China. The sampling framework included all undergraduates enrolled in public universities in the province. To ensure representativeness, we stratified the sampling process by academic year and one’s major with classes as the primary sampling units. We randomly selected classes within each stratum. The study was approved by the Ethics Board of Northwest Normal University. Before the experiment, all participants gave informed written consent in accordance with the Code of Ethics of the World Medical Association (Declaration of Helsinki).

A total of 630 questionnaires was distributed, of which 578 were valid, resulting in a response rate of 91.75%. There were 222 males (*Mage* = 21 years, *SD* = 2.06 years) and 356 females (*Mage* = 21 years, *SD* = 2.08 years). As for residential background, 38.58% (*n* = 223) of the participants were from towns, whereas 61.42% (*n* = 355) were from rural areas. With the approval and collaboration of the teaching authorities, the researcher gathered data during students’ break times (approximately 15 min) in their classrooms. Before administering the questionnaire, the researcher provided a detailed explanation of the study’s purpose, emphasizing voluntary participation and strict anonymity. The questionnaires were distributed in a group setting, with completion and collection occurring on site to maximize the response rate and data quality. We used a structured questionnaire to collect data on demographic traits, psychological attributes, and behavioral tendencies. The full content of the questionnaire is available 10.6084/m9.figshare.28022915.v1.

### Materials

#### Personality traits

The personality test is based on the Big Five Personality Questionnaire (NEO-FFI)^26^. The Chinese version was translated by Yao^34^; it contains 60 items divided into five dimensions: neuroticism, extroversion, openness, agreeableness, and conscientiousness. Each dimension has 12 items, which are rate on a 5-point scale. The reliability of this version is consistent with that of the full version and has good convergent validity with other personality measurement tools. The internal validity (*α* = 0.71) and results of confirmatory factor analysis (CFA) are good (*χ*^2^/*df* = 2.364, CFI = 0.991, TLI = 0.971, SRMR = 0.020, RMSEA = 0.049).

#### Family dynamics

Epstein et al.^13^ developed the Family Function Assessment Scale based on the McMaster Family Function (MMFF) to gauge family dynamics. The Chinese version was translated by Liu^35^. There are 60 items; all items are scored on a 4-point scale where 1 = *healthy* and 4 = *unhealthy*. We used the average score for each item on each subscale as the subscale score. The higher the score, the worse the outcome of the item evaluation and the worse the corresponding family dynamics. The scale consists of seven subscales: communication, problem-solving, affective responsiveness, affective involvement, roles, behavioral control, and general functioning. We divided the average score of each subscale item by the subscale score. The internal validity (*α* = 0.90) and results of CFA are good (*χ*^2^/*df* = 2.82, RMSEA = 0.056, SRMR = 0.020, NNFI = 0.98, IFI = 0.99, CFI = 0.99).

#### Self-control

We measured internal control using the Self-Control Scale (SCS)^33^. The Chinese version was translated by Tan and Guo^36^. We scored a total of 19 items—including the five dimensions of impulse control, health habits, resistance to temptation, focused learning, and control of entertainment—on a 5-point scale, and we scored some items in reverse. The scale has good reliability and validity (*α* = 0.82). The CFA indicates a good fit (*χ*^2^/*df* = 1.63, RMSEA = 0. 033, SRMR = 0.018, NNFI = 0.99, IFI = 0.99, CFI = 0.99).

#### Impulsive buying

We measured IB using the Chinese Consumer Impulse Purchasing Tendency Scale^37^. The internal consistency is good (*α* = 0.90). The participants rated the extent to which they agreed with the statements (e.g., “If you have money today, you can spend it today, and if you have money tomorrow, you can spend it tomorrow”) on a 7-point scale, with 1 = *totally disagree* and 7 = *totally agree*. The CFA suggests a good fit (*χ*^2^/*df* = 3.98, RMSEA = 0.072, SRMR = 0.018, NNFI = 0.97, IFI = 0.99, CFI = 0.99).

### Statistical analysis

We employed SPSS 22.0 to perform an independent samples *t*-test and correlation analysis. We used AMOS 21.0 for CFA and to build the structural equation model, and we utilized bootstrapping to carry out an indirect effect test.

## Results

### Testing for common method bias

We only used a self-report method to collect data; thus, there may have been common method bias (CMB). To further improve the rigor of the study, we utilized Harman’s single-factor test to establish the existence of CMB before analyzing the data^38^; 51 factors had eigenvalues greater than 1, while the first factor of variance explained 18.51% less than the critical value of 40%. Thus, our study does not have severe CMB.

### Correlation analysis among the variables

Table 1 outlines the correlation analysis of the personality traits and other potential variables. Neuroticism is positively correlated with family dynamics and IB but negatively correlated with self-control (*p* < 0.05). Extroversion, agreeableness, and conscientiousness are negatively correlated with family dynamics and IB, and positively correlated with self-control (*p* < 0.05). Openness is not significantly correlated with self-control and IB (*p* > 0.05). Family dynamics is positively correlated with self-control and IB (*p* < 0.05).

**Table 1:** Relevant analysis of personality traits and other latent variables.

	M ± SD	*N*	E	A	C	O	FAD	SCS	IB
N	2.86 ± 0.59	1							
E	3.31 ± 0.50	-0.46^**^	1						
A	3.34 ± 0.44	-0.32^**^	0.32^**^	1					
C	3.44 ± 0.50	-0.40^**^	0.45^**^	0.37^**^	1				
O	3.44 ± 0.50	-0.12^**^	-0.25^**^	0.12^**^	0.28^**^	1			
FAD	2.21 ± 0.30	0.33^**^	-0.31^**^	-0.34^**^	-0.42^**^	-0.32^**^	1		
SCS	3.21 ± 0.51	-0.40^**^	0.23^**^	0.33^**^	0.56^**^	0.08	-0.34^**^	1	
IB	3.33 ± 0.86	0.16^**^	-0.12^**^	-0.33^**^	-0.34^**^	-0.07	0.32^**^	-0.50^**^	1

N = neuroticism; E = extroversion; A = agreeableness; C = responsibility; O = openness; SCS = total score of self-control; FAD = total score of family function; IB = total score of impulsive buying, *p < 0.05. **p < 0.01, the same below.

### Mediation test

**Fig. 2:**
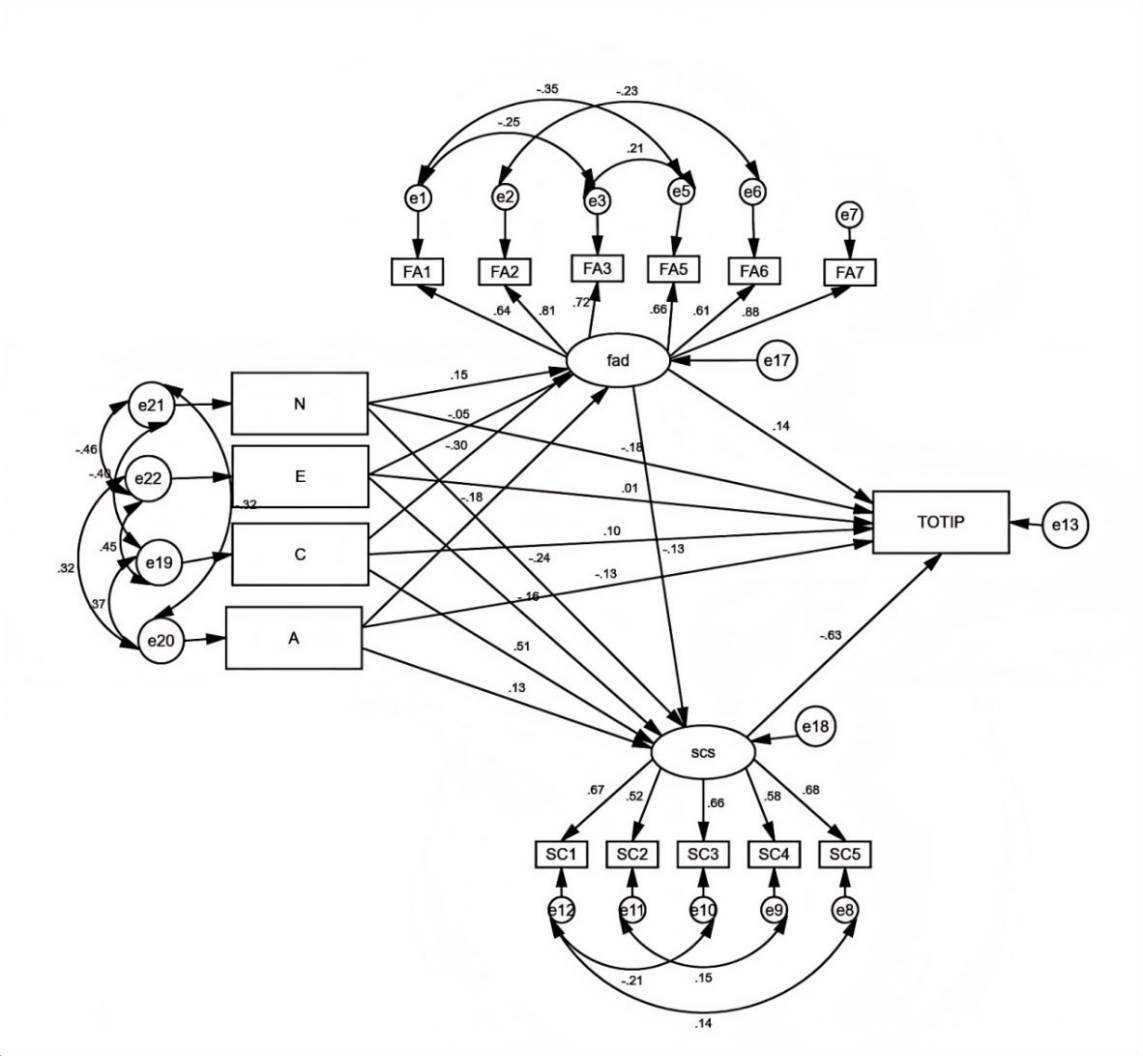
AMOS results output figure. *Notes* N = neuroticism; E = extroversion; A = agreeableness; C = responsibility; FAD = total score of family function; FA1 = problem solving; FA2 = communication; FA3 = roles; FA4 = affective responsiveness; FA5 = affective involvement; FA6 = behavior control; FA7 = general functioning; SCS = total score of self-control; SC1 = impulse control; SC2 = health habits; SC3 = resist temptation; SC4 = focus on learning; SC5 = controlled entertainment; TOTIP = total score of impulse consumption.

According to Wen’s process for testing the mediation effect, we employed AMOS to analyze the relationship between personality traits, family dynamics, self-control, and IB^39^. We used bootstrapping with 1,000 samples and a 95% confidence interval (CI) to perform a mediation test; if the CI does not contain 0, this indicates a significant mediating effect. The analysis of the structural equation model shows a good model fit, *χ*^*2*^ = 250, *df* = 87, *χ*^*2*^*/df* = 3.094, RMSEA = 0.060, SRMR = 0.042, NNFI = 0.93, ILI = 0.95, CFI = 0.95. Figure 2 showed AMOS results output figure.

#### Personality traits and IB

Figure 2 indicates that neuroticism and agreeableness have significant, negative predictive effects on IB (*β* = -0.18, *p* < 0.01; *β* = -0.13, *p* < 0.01), while extroversion and conscientiousness have no significant predictive effects on IB (*β* = 0.01, *p* > 0.05; *β* = 0.1, *p* > 0.05). Thus, H1 is supported.

#### Mediation test of family dynamics between personality traits and IB

**Fig. 3:**
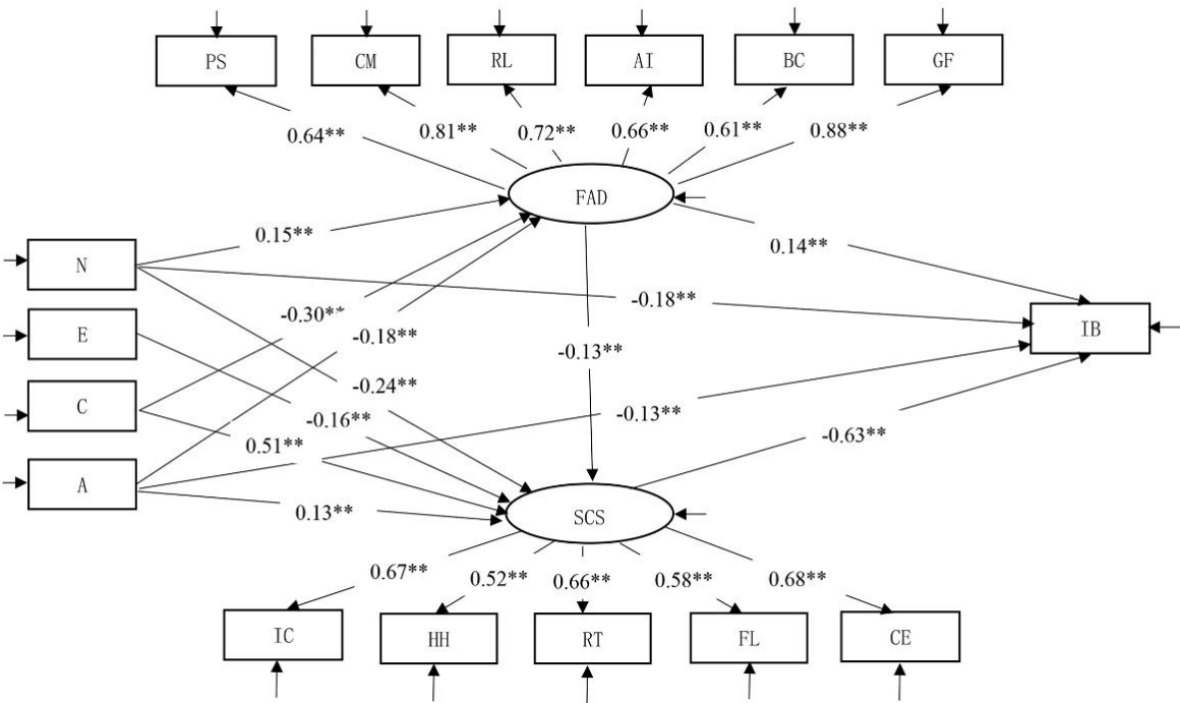
Chain mediation model between variables. *Notes* PS = problem solving; CM = communication; RL = roles; AR = affective responsiveness; AI = affective involvement; BC = behavior control; GF = general functioning; IC = impulse control; HH = health habits; RT = resist temptation; FL = focus on learning; CE = controlled entertainment.

Figure 3 suggests that neuroticism has significant, positive predictive effects on family dynamics (*β* = 0.15, *p* < 0.01), while conscientiousness and agreeableness have significant, negative predictive effects on family dynamics (*β* = -0.30, *p* < 0.01; *β* = -0.18, *p* < 0.01). Family dynamics have significant, positive predictive effects on IB (*β* = 0.14, *p* < 0.01). We used bootstrapping with a 95% CI. The indirect effect value (IEV) of family dynamics in the influence of neuroticism on IB is 0.02, with a 95% CI [0.005, 0.045]. The IEV of family dynamics in the influence of agreeableness on IB is -0.03, with a 95% CI [-0.147, -0.018]. The IEV of family dynamics in the influence of conscientiousness on IB is -0.04, with a 95% CI [-0.078, -0.012]. Hence, H2 is supported.

#### Mediation test of self-control between personality traits and IB

Figure 2 indicates that neuroticism and extroversion have significant, negative predictive effects on self-control (*β* = -0.24, *p* < 0.01; *β* = -0.16, *p* < 0.01). Conscientiousness and agreeableness have significant, positive predictive effects on self-control (*β* = 0.51, *p* < 0.01; *β* = 0.13, *p* < 0.01). Self-control has significant, negative predictive effects on IB (*β* = -0.63, *p* < 0.01). The IEV of self-control in the influence of neuroticism on IB is 0.15, with a 95% CI [0.088, 0.228]. The IEV of self-control in the influence of agreeableness on IB is -0.08, with a 95% CI [-0.147, -0.018]. The IEV of self-control in the influence of conscientiousness on IB is -0.32, with a 95% CI [-0.425, -0.226]. Thus, H3 is supported. The relationship between extroversion and IB is fully mediated by self-control (95% CI [0.035, 0.172]). Thus, H3 is supported.

#### The chain mediation test of family dynamics and self-control

Figure 2 shows that family dynamics have significant, negative predictive effects on self-control as it relates to family dynamics (*β* = -0.13, *p* < 0.01). The IEV of family dynamics and self-control in the influence of neuroticism on IB is 0.01, with a 95% CI [0.0004, 0.053]. The IEV of family dynamics and self-control in the influence of agreeableness on IB is -0.01, with a 95% CI [-0.056, -0.001]. The IEV of family dynamics and self-control in the influence of conscientiousness on IB is -0.03, with a 95% CI [-0.088, -0.001]. Thus, H4 is supported. The results are shown in Table 2.

**Table 2:** Mediation path analysis results.

	Indirect effect value	Bootstrap 95% confidence interval	Relative mediating effect
Total indirect effect between N-IB	0.19	[0.128, 0.264]	51.35%
FAD	0.02	[0.005, 0.045]	5.41%
SCS	0.15	[0.088, 0.228]	40.54%
FAD-SCS	0.01	[0.000^a^, 0.053]	2.70%
Total indirect effect between A-IB	-0.12	[-0.195, -0.065]	48.00%
FAD	-0.03	[-0.049, -0.007]	12.00%
SCS	-0.08	[-0.147, -0.018]	32.00%
FAD-SCS	-0.01	[-0.056, -0.001]	4.00%
Total indirect effect between C-IB	-0.39	[-0.524, -0.292]	79.59%
FAD	-0.04	[-0.078, -0.012]	8.16%
SCS	-0.32	[-0.425, -0.226]	65.31%
FAD-SCS	-0.03	[-0.088, -0.001]	6.12%
Total indirect effect between E-IB	0.09	[0.031, 0.174]	90.69%
SCS	0.10	[0.035, 0.172]	90.00%

N = neuroticism; E = extroversion; A = agreeableness; C = responsibility; O = openness; SCS = total score of self-control; FAD = total score of family function; IB = total score of impulsive buying.

^a^ is 0.0004. Reserving three decimal places is about 0.000.

## Discussion

### The impact of personality characteristics on IB

Openness, extroversion, and neuroticism are positively correlated with IB, whereas conscientiousness and agreeableness are negatively correlated with it^40,41^. Further, individuals with high levels of neuroticism are more responsive to external stimuli and experience more interpersonal problems and stress^29^. Neuroticism is negatively correlated with IB, possibly because IB fails to address underlying issues; this may have become an ineffective coping mechanism over time. As for agreeableness, our findings align with those of earlier studies^42^, suggesting that greater levels of agreeableness are associated with reduced IB. Individuals with high agreeableness prioritize harmonious relationships, possess strong interpersonal skills, and are more inclined to consider others’ perspectives, which encourages thoughtful decision-making and reduces IB behavior.

### The mediating role of family dynamics between personality traits and IB

This study suggests that family dynamics mediate the relationship between personality traits and IB. Neuroticism positively predicts IB through poor family dynamics, whereas conscientiousness and agreeableness negatively predict IB through better family dynamics. These findings align with previous research showing that neurotic individuals, due to their heightened sensitivity to negative stimuli and frequent experiences of negative emotions, may adopt negative problem-solving approaches and communication patterns to handle family relationships, further leading to passive coping strategies (such as IB) to alleviate stress and fulfill internal needs^8,43^. Conversely, positive personality traits such as conscientiousness and agreeableness are associated with harmonious family ties, effective communication, and constructive problem-solving, which reduces the likelihood of impulsive behavior^44^. We did not find a relationship between extroversion and family dynamics; nor did family dynamics mediate the relationship between extroversion and IB. Individuals with high extroversion are sociable, enthusiastic, and confident^45^, which is consistent with the findings of Ratnawat and Borgave^20^. Individuals with high extroversion are more proactive in maintaining family relationships, enjoy communicating, and are more likely to achieve good family dynamics, thereby avoiding IB. Additionally, the impulsive consumption of extroverted individuals is susceptible to factors such as purchasing scenario and gender^20^. Future research should explore the combined effects of these factors and family dynamics on IB.

Expanding on earlier studies, our results emphasize the dual role of family dynamics as both an influence on personality traits and as a predictor of external behavior, demonstrating the critical role of family dynamics as an intermediary. While prior research has primarily focused on the direct relationship between personality traits and impulsive behavior^46^, this study underscores the importance of external environmental factors, such as family dynamics, in shaping such behavior^24^.

### The mediating role of self-control between personality traits and IB

The present study indicates that self-control serves as a mediating factor in the relationship between personality traits and IB. Specifically, conscientiousness and agreeableness negatively predict IB through self-control, whereas neuroticism and extroversion positively predict IB through the same mechanism. These findings align with prior research identifying self-control as a key determinant in moderating or facilitating impulsive behavior^46,47^. Agreeableness indicates a caring, compassionate, helpful, and sincere attitude. Conscientiousness refers to the awareness of fulfilling responsibilities in an orderly manner^20^. Conscientiousness and self-control emphasize goal-oriented behavior and responsible decision-making, thereby enhancing self-control and reducing IB^48^. Individuals with high neuroticism and extroversion tend to have more intense emotional experiences, which can impact self-control and lead to impulsive behavior^48^.

Previous studies have shown that self-control influences impulsive behavior by regulating responses to external temptation. For instance, low self-control directly correlates with IB and fosters positive attitudes toward targeted ads and impulsive tendencies on social networks, thereby amplifying IB behavior^49^. Our findings extend the work of Roberts et al.^50^, who established self-control as a pivotal mediating factor in impulsive and compulsive buying. Moreover, we derived nuanced results regarding neuroticism. While neuroticism is typically associated with IB due to heightened emotional responses and reduced self-regulation^29^, our findings imply that this relationship can shift depending on the mediating factors. Specifically, when self-control mediates this relationship, the positive effect of neuroticism on IB is mitigated, illustrating that self-control can reshape the behavioral outcomes of neurotic tendencies. This finding diverges from the results of earlier studies, which primarily emphasize the direct positive effects of neuroticism on IB. Hence, the present study offers a novel perspective on how personality traits interact with regulatory mechanisms.

### The chain mediation of family dynamics and self-control

Families play an important role in shaping individual personality and the self, and have a profound impact on physical and mental development^51^. We found that family dynamics has a chain-mediating effect on the process of personality traits influencing IB. Family dynamics indirectly impact IB by influencing self-control. While previous research has highlighted the significant role of family dynamics in behavioral regulation^52^, our results imply that self-control exerts a stronger, more direct influence on IB. When family dynamics and self-control operate together, the regulatory effects of family dynamics are fairly weak. The phenomenon described in mental accounting theory^52^ illustrates how individuals’ spending behavior differs based on the perceived ownership of funds, with less self-regulation when money is viewed as “extra” or “free” (e.g., spending paid for by one’s parents). This finding reinforces the notion that self-control, as an intrinsic ability, has a more robust impact on IB, especially when external influences (such as family dynamics) are present.

Our study reveals a complex interaction between neuroticism, family dynamics, and self-control. Although there is a positive correlation between neuroticism and IB, in the direct effect of the structural equation model, neuroticism negatively predicts IB. Some studies suggest that the total effect (*c*) between the two variables is not significant, but there may still be indirect effects^53^. For example, the indirect effect is opposite to the symbol for the direct effect, and there are two mediating paths whose effects are similar in size but opposite in direction. In this study, the symbols for the direct and indirect path coefficients are opposite, but due to the significant direct effect, the influence of family dynamics and self-control on neuroticism and IB does not conform to the suppressing effect. Family dynamics and self-control are two mediating variables with similar effects and opposite directions. In this study, when we added family dynamics and self-control, the positive predictive effect of neuroticism and IB changed to a negative predictive effect, thus altering the path symbols. Specifically, family dynamics and self-control are key variables impacting neuroticism and IB; this modifies the relationship between neurotic personality traits and IB behavior. This shows that in a healthy family environment, individuals can reduce the congenital genetic influence from families and individuals, thereby decreasing negative behaviors (such as IB) by exercising self-control.

Our results also highlight the context in which family dynamics indirectly weaken the effect of self-control on IB. This finding contrasts with the results of other studies, which suggest that self-control is the dominant mediator in regulating impulsive behavior^49,50^. For example, individuals with greater self-control may exhibit IB behavior when family dynamics provide inconsistent or conflicting cues about spending, such as permissive parenting practices. These results illustrate the nuanced interplay between external family influences and internal regulatory mechanisms.

## Findings and limitations of the study

### Theoretical and practical findings

This study contributes significantly to the theoretical understanding of the mechanisms underlying IB by integrating personality traits, family dynamics, and self-control into a comprehensive framework. It confirms the established relationships between personality traits and impulsive behavior, in addition to expanding upon them by revealing the mediating roles of family dynamics and self-control. The findings underscore how external environmental factors (such as family dynamics) and internal regulatory mechanisms (such as self-control) interact to shape IB. This integrative view addresses gaps in the existing literature, which often considers these factors in isolation, thereby advancing theoretical models of consumer behavior and psychological regulation. A novel theoretical contribution of this study is the identification of the chain-mediating effect of family dynamics and self-control, especially in the context of personality traits such as neuroticism. By demonstrating how a supportive family environment and enhanced self-control can mitigate the behavioral consequences of neuroticism, this study challenges traditional views that emphasize the immutability of personality-driven behaviors. Our study provides evidence for the modifiability of these pathways, offering a dynamic perspective on the interaction between personality traits and external influences.

The findings offer practical applications for educators, mental health practitioners, and policymakers by providing actionable strategies for addressing IB. In educational settings, particularly universities, fostering self-control among students is critical as programs focused on financial literacy and self-regulation can effectively help students manage their spending behaviors. For example, workshops on budgeting and resisting marketing temptations can mitigate IB, particularly in contexts where students manage their finances independently. For mental health practitioners and counselors, this study underlines the pivotal role of family dynamics in shaping behavioral outcomes. Strengthening family communication and support systems can build environments conducive to developing positive personality traits and reducing impulsive tendencies. Family-based interventions that address dysfunctional family patterns or encourage emotional support can indirectly enhance self-control, further reducing maladaptive behaviors such as IB. Our findings also demonstrate the importance of policymakers and marketers balancing commercial incentives with ethical considerations. Policymakers can advocate for regulations that limit targeted advertising aimed at vulnerable groups (such as financially inexperienced university students), while marketers can design campaigns that promote long-term value and delayed gratification (versus immediate rewards). Collectively, these strategies aim foster financial well-being and behavioral resilience among individuals vulnerable to IB.

### Limitations

This study has some limitations. First, it is a cross-sectional study; thus, it was impossible for us to draw causal inferences based on the results. Any causal relationship between the variables needs to be further examined and verified by combining experiments and tracking studies to reveal the mechanism of action between the variables more deeply. Second, we obtained the data from the self-reports of college students. As such, there may have been a socially appreciative effect. Individuals try to shape the image of social expectations or evade punishment and falsely report the degree of individual negative behavior. Future research should consider multi-agent assessments to obtain more objective and comprehensive information.

## Data Availability

The data presented in this study are available on request from the corresponding author.
